# Don't Let the Truth Get in the Way of a Good Story: An Illustration of Citation Bias in Epidemiologic Research

**DOI:** 10.1093/aje/kwu164

**Published:** 2014-07-02

**Authors:** Mika Kivimäki, G. David Batty, Ichiro Kawachi, Marianna Virtanen, Archana Singh-Manoux, Eric J. Brunner

**Affiliations:** 1 Department of Epidemiology and Public Health, University College London, London, United Kingdom; 2 Hjelt Institute, Medical Faculty, University of Helsinki, Helsinki, Finland; 3 Centre for Cognitive Ageing and Cognitive Epidemiology and Alzheimer Scotland Dementia Research Centre, University of Edinburgh, Edinburgh, United Kindom; 4 Department of Society, Human Development, and Health, Harvard School of Public Health, Boston, MA; 5 Finnish Institute of Occupational Health, Helsinki, Finland; 6 INSERM U1018, AP-HP, Villejuif, France

The production of scientific knowledge is susceptible to bias at every stage of the process, from what questions are asked by the investigator, to which method is chosen to gather data, to which analyses are conducted (e.g., “P-hacking,” wherein the method of statistical analysis and the degrees of freedom are manipulated until they yield statistically significant results) (1). Even after completion of a study, authors sometimes choose not to submit their work for publication because they are not satisfied with the results (i.e., the “file drawer” problem) (1), or they encounter difficulties with getting results published because of reviewer or editorial bias (“publication bias”) (2–4).

Although prepublication biases have been well described in epidemiologic textbooks, postpublication biases, such as selective citation, have been less well documented. “Citation bias” occurs when scientists selectively cite papers based upon risk estimates that conform to their preconceived notions (5). When researchers have a bias in favor of “X causing Y,” they are more likely to cite papers that found evidence to support their view. Conversely, when researchers harbor a bias against a hypothesized association, they may selectively cite papers that report null findings.

Here, we use research on job strain and the risk of coronary heart disease to examine factors that influence citations in peer-reviewed literature. In addition to the risk estimate for job strain relative to no job strain in each study, we take into account the impact factor of the publishing journal, which is an indicator of its prestige.

## METHODS

We used the most recent meta-analysis of job strain and incident coronary heart disease to identify relevant studies for this analysis (6). According to the total evidence from this meta-analysis (26 studies), employees who experienced job strain had 1.34 (95% confidence interval (CI): 1.18, 1.51) times greater disease risk than did those free of job strain (6). To allow an adequate period of time for citations to accumulate, we focused on papers published at least 10 years ago, which yielded a total of 7 cohort studies (Table 1) (7–13). For each study, we obtained relative risk estimates for the job strain–heart disease association, counted citations in the Scopus (Elsevier, Amsterdam, the Netherlands) and Web-of-Science (Thompson Reuters, New York, New York) databases, and obtained the impact factor of the publishing journal from Web-of-Science Journal Citations 2013. In addition, we obtained an indicator of the scientific quality of each study from a review (14) in which the authors had based their evaluation on 8 criteria (e.g., the characteristics of the study population, validity of the exposure measurement and outcome ascertainment, and comprehensiveness of adjustments for confounding factors). A higher score indicated higher quality (range, 0–12) (14).

**Table 1.** Number of Citations, Effect Size, Journal Impact Factor, and Scientific Quality for Cohort Studies on Job Strain and Coronary Heart Disease Published From 1989 to 2004

First Author, Year (Reference No.)	No. of Citations^a^		Relative Risk	95% CI	Journal Impact Factor^b^	Quality Score^c^
	Scopus	Web-of-Science				
Kivimäki, 2002 (11)	384	328	2.20	1.16, 4.17	17.215	4
Johnson, 1989 (8)	255	252	1.94	1.15, 3.21	3.775	6
Kuper, 2003 (12)	219	203	1.57	1.26, 1.96	3.393	
Eaker, 1992 (13)	200	189	0.94	0.45, 1.44	4.780	8
Reed, 1989 (7)	125	147	0.94	0.65, 1.36	4.780	6
Alterman, 1994 (9)	120	116	1.48	0.98, 2.24	4.780	8
Lee, 2002 (10)	79	70	0.80	0.48, 1.33	6.982	8

Abbreviation: CI, confidence interval.

^a^ Citations as of January 25, 2014.

^b^ Web of Science journal impact factor for 2013.

^c^ Quality score (range, 0–12) was obtained from a previous review (14). The quality score for Kuper et al. (12) is missing because it was not included in that review.

We computed the associations of effect size and journal impact factor with the number of citations using general linear models (procedure glm in Stata, version 11.2; StataCorp LP, College Station, Texas). Both analyses were adjusted for citation-time (the time period during which the study could have been cited).

## RESULTS

The total number of citations for the 7 studies was 1,382 in Scopus and 1,305 in Web-of-Science. In the most-cited paper, job strain was reported to double the disease risk (Table 1) (11). In contrast, the least-cited paper reported a null finding (10). There was a general trend toward higher frequencies of citations for papers that reported a higher risk ratio (7–13). After controlling for citation-time, we found that each 10% increase in reported excess risk was associated with 16.5 (95% CI: 7.9, 25.0) additional citations in Scopus (*P* = 0.001, adjusted *R*^2^ = 0.69) and 12.4 (95% CI: 1.1, 23.7) additional citations in Web-of-Science (*P* = 0.03, adjusted *R*^2^ = 0.31).

Moreover, a higher journal impact factor was associated with a higher citation frequency, although this was imprecisely estimated, as evidenced by the wide confidence intervals (in Scopus, per each 1-point increase in impact factor, change in citations = 13.9, 95% CI: −2.8, 30.6, *P* = 0.10, adjusted *R*^2^ = 0.15; in Web-of-Science, change in citations = 14.3, 95% CI: 0.5, 28.0, *P* = 0.04, adjusted *R*^2^ = 0.27).

High-quality evidence was seemingly not a priority when authors decided which articles to cite. The most-cited study had the lowest scientific quality score (4) of all papers (11). In contrast, the 2 least-cited studies obtained a quality score of 8, which was the highest received (9, 10).

## DISCUSSION

By analyzing the frequency of citation of papers that examined the relation of job strain with coronary heart disease, we showed that higher-quality science in this field did not garner more citations. In contrast, studies that reported higher risk estimates were cited more frequently than those that reported lower risk estimates. Similarly, as described elsewhere (5), there was a tendency for articles that were published in the more prestigious journals to be cited more often.

A strength of the present analysis is that we targeted research on a specific topic. This facilitated a straightforward comparison between studies. Study quality was determined based on a score obtained from an independent review (14); unfortunately, this score was missing for one of the target papers (12).

The main limitation of our investigation is the small number of studies included in the analyses (7–13). Our findings should therefore be interpreted in this context; it is unknown whether they are generalizable to other areas of epidemiology. More general limitations of examining citations as an outcome include the fact that citation bias may be bi-directional; for example, tobacco industry–funded researchers may be motivated to cite studies that found null associations between smoking and disease. Further, a citation could be included in a critical context or as counterfactual evidence.

However, our findings are in agreement with those from previous studies. In an examination of citations of published articles that were originally submitted to an emergency medicine specialty meeting, Callaham et al. (2) found that the strength of methodology and study design did not predict the frequency of citations during a 3.5-year follow-up. Positive outcome bias was not observed either, but the constituent studies were focused on a heterogeneous set of topics (2). Jannot et al. (5) retrieved citation counts of specific therapeutic intervention studies and found that studies with statistically significant findings were cited twice as often as those with nonsignificant findings. Similarly, Andrade et al. (15) reported that trials that reported favorable outcomes for surgery to alleviate chronic nonspecific low back pain tended to be cited more often than those that reported less favorable results.

Selective publication and citation can influence the construction of scientific knowledge and even lead to nonoptimal choices for prevention. Researchers as well as journal reviewers and editors need to pay more attention to these biases. As the number of large-scale individual-participant meta-analyses and the amount of data sharing increase within the research community, epidemiology as a science can become more transparent and self-critical.
